# Effectiveness of coral relocation as a mitigation strategy in Kāne‘ohe Bay, Hawai‘i

**DOI:** 10.7717/peerj.3346

**Published:** 2017-05-23

**Authors:** Ku’ulei S. Rodgers, Koi Lorance, Angela Richards Donà, Yuko Stender, Claire Lager, Paul L. Jokiel

**Affiliations:** 1 University of Hawai‘i, Hawai‘i Institute of Marine Biology, Kāne‘ohe, HI, United States of America; 2 Taylor Shellfish Natural Energy Laboratory, Kailua-Kona, HI, United States of America

**Keywords:** Coral reef, Dredging, Restoration, Hawai‘i, Transplantation, Coral relocation

## Abstract

Coral reef restoration and management techniques are in ever-increasing demand due to the global decline of coral reefs in the last several decades. Coral relocation has been established as an appropriate restoration technique in select cases, particularly where corals are scheduled for destruction. However, continued long-term monitoring of recovery of transplanted corals is seldom sustained. Removal of coral from a navigation channel and relocation to a similar nearby dredged site occurred in 2005. Coral recovery at the donor site and changes in fish populations at the receiving site were tracked periodically over the following decade. Coral regrowth at the donor site was rapid until a recent bleaching event reduced coral cover by more than half. The transplant of mature colonies increased spatial complexity at the receiving site, immediately increasing fish biomass, abundance, and species that was maintained throughout subsequent surveys. Our research indicates that unlike the majority of historical accounts of coral relocation in the Pacific, corals transplanted into wave-protected areas with similar conditions as the original site can have high survival rates. Data on long-term monitoring of coral transplants in diverse environments is central in developing management and mitigation strategies.

## Introduction

Natural and anthropogenic damage to coral reefs is rapidly accelerating worldwide (Pandolfi et al., 2003). These acute and chronic threats include local impacts such as pollution, sedimentation, eutrophication, ship groundings, invasive species, fishing pressure, coastal development, and anchor damage that can cause severe degradation. In addition, global stressors due to rising emissions of greenhouse gases (IPCC, 2014) are leading to increased ocean temperature, ocean acidification, and storm activity that have substantially reduced coral cover on many reefs. The 1998 El Niño-Southern Oscillation (ENSO) warming event devastated 40% of the world’s coral reefs (Wilkinson, 2004; Precht, 2006) and the most recent ENSO (2014–2016) has had an even greater negative impact in many locations.Annual bleaching events can be expected on the majority of tropical reefs before the end of the century (IPCC, 2007; Hoegh-Guldberg, 1999) and the need for reef restoration will continue to grow.

Restoration and mitigation options include indirect management measures to remove obstacles to natural recovery as well as direct mediation such as coral transplantation. Coral reef transplantation efforts have traditionally been focused on repairing or replacing coral loss caused by destructive local impacts. Coral transplantation has greatly increased in the past two decades in numerous locations across the globe, e.g., Philippines, Singapore, Thailand, Mauritius, Tanzania, Seychelles, Maldives, Jordan, Israel, Hawai‘i, Japan, Taiwan, Mexico, Puerto Rico, Jamaica, Colombia, Belize, Florida Keys, Costa Rica, and others (Rinkevich, 2014). Degraded reef restoration and coral relocation due to coastal development and/or dredging, are among the most common reasons for transplantation. Corals that are transplanted onto a degraded reef generally come from dedicated coral nurseries (Rinkevich, 2014; Kotb, 2016; Montoya-Maya et al., 2016) or are directly fragmented from separate donor colonies (Guzman, 1991) while dredging or harbor construction more commonly result in relocation of entire colonies. Broken fragments may also be attached directly to natural and artificial substrates within the same reef areas (Tortolero-Langarica, Cupul-Magaña & Rodríguez-Troncoso, 2014). Substrate at the receiving site may be natural reef, rubble, or sand, or in some cases, artificial structure may be necessary (Muñoz Chagín, 1997; Kotb, 2016). Reef repair can be costly and futile if the factors causing the damage are not removed and restoration efforts cannot be evaluated without long-term monitoring of the mitigation effort. Such documentation has often been neglected due to lack of legislative requirements, funding, or accessibility (Precht, 2006), however, that is changing and more and more research on this topic is yielding positive outcomes for reefs (Horoszowski-Fridman & Rinkevich, 2015).

Previous coral reef transplantation in the Pacific has been summarized by Jokiel et al. (2006) and recommendations for undertaking such activity have been published by Naughton & Jokiel (2001). They conclude corals are often transplanted into marginal habitats with initial success but eventual decline is attributed to wave damage, sedimentation, and/or eutrophication. Research on Kāne‘ohe Bay dredged reefs was conducted by Uchino (2004) who compared fish and coral populations on a dredged versus an undredged patch reef. The dredged reef failed to recover substantially over the past 60 years, although environmental conditions for coral growth at that location are highly favorable except for the presence of a sandy mud substrate that has blocked new coral recruitment. Coral larvae cannot settle and grow on soft substrate, but transplanted large coral colonies can do extremely well under these conditions (Perez III et al., 2014; Jokiel et al., 2014).

Successful coral reef restoration has previously been accomplished in Kāne‘ohe Bay (e.g., Jokiel & Brown, 1998; Jokiel et al., 1999). Many of the coral reefs there were severely damaged between 1937 and 1944 by dredge and fill operations undertaken to create ship channels and seaplane runways during construction of the Kāne‘ohe Naval Air Station (Devaney, 1976). Maragos (1974) demonstrated that transplantation of corals is a viable technique for restoring reefs in Kāne‘ohe Bay. Kolinski & Jokiel (1996) moved corals destined to be destroyed by dredging from the Kāne‘ohe Bay Yacht Harbor to reef that was dredged circa 1938 at the east end of the Bay. The transplanted corals had >90% survival and effectively transformed the dredged sandy area into a functional coral reef.

**Figure 1:**
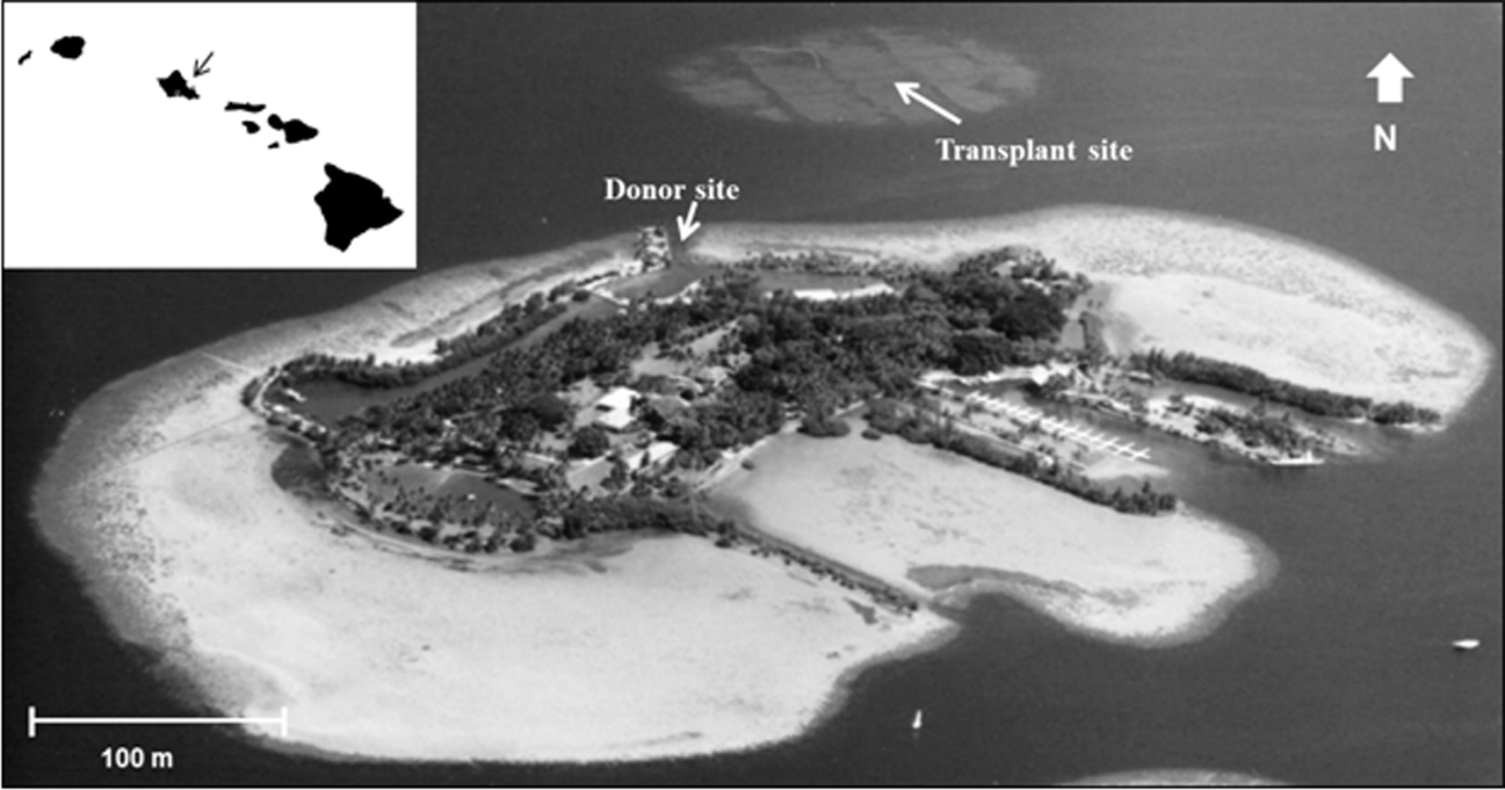
Aerial photograph showing location of the donor site (channel) and transplant site for corals in south Kāne‘ohe Bay, O‘ahu, Hawai‘i. Image: Hawai‘i Institute of Marine Biology.

During 2005 the opportunity arose to expand previous reef restoration work in Kāne‘ohe Bay, based on established procedures. Corals needed for this restoration became available through a project directed at removing navigation obstructions in the channel leading to the University of Hawai‘i’s Hawai‘i Institute of Marine Biology (HIMB) at Moku o Lo‘e Island (Fig. 1). During the past 60 years corals in the shallow entrance channel in front of the laboratory grew to a considerable size obstructing vessel passage through the channel at low tide. Removal of these large coral heads was necessary and a nearby dredged patch reef was selected as the receiving site (Fig. 1). Moving of the corals to the restoration site was accomplished with assistance of U.S. Army dive and salvage teams who undertook the mission as part of their ongoing training program. A three to five year period is recommended for adequate ecosystem stabilization due to the slow growth of corals (Precht, 2006) thus subsequent resurveys at both the donor and relocation sites were conducted in 2008, 2012, and 2016 to determine changes in fish and coral populations.

## Materials and Methods

Relocation site selection was based on proximity to donor site, adequate depth to avoid creation of a navigational hazard, and presence of a large area of dredged sand substrate, which lacked coral cover. Donor site depth ranged from 0.5 m-2 m depth while corals were placed at the relocation site at a depth of approximately 3–4 m. Receiving locations were marked with a subsurface buoy and documented using GPS and triangulation lineups. Corals blocking the main navigation channel (Fig. 1) were removed and transplanted to the dredged patch reef by volunteer U.S. Army divers between November 2004 and March 2005. Salvaged corals were removed from the substrate with a large pry bar and placed in carriers constructed of a stable platform of soft wire netting to protect the corals during transport. Lift bags provided neutral buoyancy for ease of movement and the carriers were suspended under inflatable vessels. The corals remained entirely submerged during their short journey to the receiving site. An estimated buoyant weight of 68,000 kg of *Porites compressa* (finger coral) and *Montipora capitata* (rice coral) were moved. This weight of corals represents an approximate 200 m^2^ map area with high vertical relief. To avoid spread of invasive species, flora and fauna at both the donor and the receiving sites were surveyed prior to relocation efforts. The invasive orange sponge *Mycale grandis* (orange-keyhole sponge) was found to be present at both sites. The shallow-water invasive algal species *Gracilaria salicornia* was not found at the receiving site, which is below its normal depth range. Although physical and biological limitations prevent the spread of the invasive alga *G. salicornia* to deeper patch reefs, extreme care was taken to exclude any attached algae. All divers were trained in recognizing and avoiding this alga. Fragment spread by divers, gear, and equipment was minimized during the operations through gear inspection and cleaning, supervision by ecologists, and inspection of corals at the receiving site. Exceptional care was taken to avoid any damage to marine life during all operations. The few corals from the inner portion of the channel that had attached *G. salicornia* were placed on the Moku o Lo‘e reef immediately outside the entrance channel in an area where this invasive, alien alga is already well established.

### Donor site

Ten permanent survey sites were strategically placed in the channel to allow full documentation of changes following transplantation. These sites were marked with stainless steel pins and coded cable ties to allow for future resurveys. Initial sites established in 2005 were resurveyed in 2008, 2012, and 2016. An Olympus 5050 camera with underwater housing attached to a monopod for stability, consistent distance from the bottom, and constant image size that covered a 50 cm × 63 cm area. Images were analyzed using PhotoGrid (Bird, 2001). Proportion of coral cover was transformed using a square-root transformation to meet the assumption of normality and equal variance for statistical tests. A General Linear Model (GLM) (MINITAB 17) was used to examine the differences in mean total coral cover between years followed by Tukey Pairwise comparisons with simultaneous 95% Confidence Intervals (CI). Percent changes (Excel 2010) were calculated to compare temporal changes in coral cover and composition between years. The differences in median cover of two coral species, *Montipora capitata* and *Porites compressa* were examined separately using a Kruskal–Wallis test.

### Relocation site

Fish populations were recorded using standard visual belt transects (Brock, 1954). Divers using SCUBA swam along one 50 m × 5 m transect (125 m^2^) recording species, abundance, and total fish length. All fishes were identified to the lowest taxon possible and estimated to the nearest centimeter. Length-weight fitting parameters obtained from the Hawai‘i Cooperative Fishery Research Unit and Fishbase (http://www.fishbase.org) were used to convert total length to biomass (Kg m^−2^). Trophic levels for fish species were determined using published data. This research was conducted under the Hawai‘i Department of Land and Natural Resources-Division of Aquatic Resources Special Activity Permit No. 2005-25.

## Results

### Donor site

**Figure 2:**
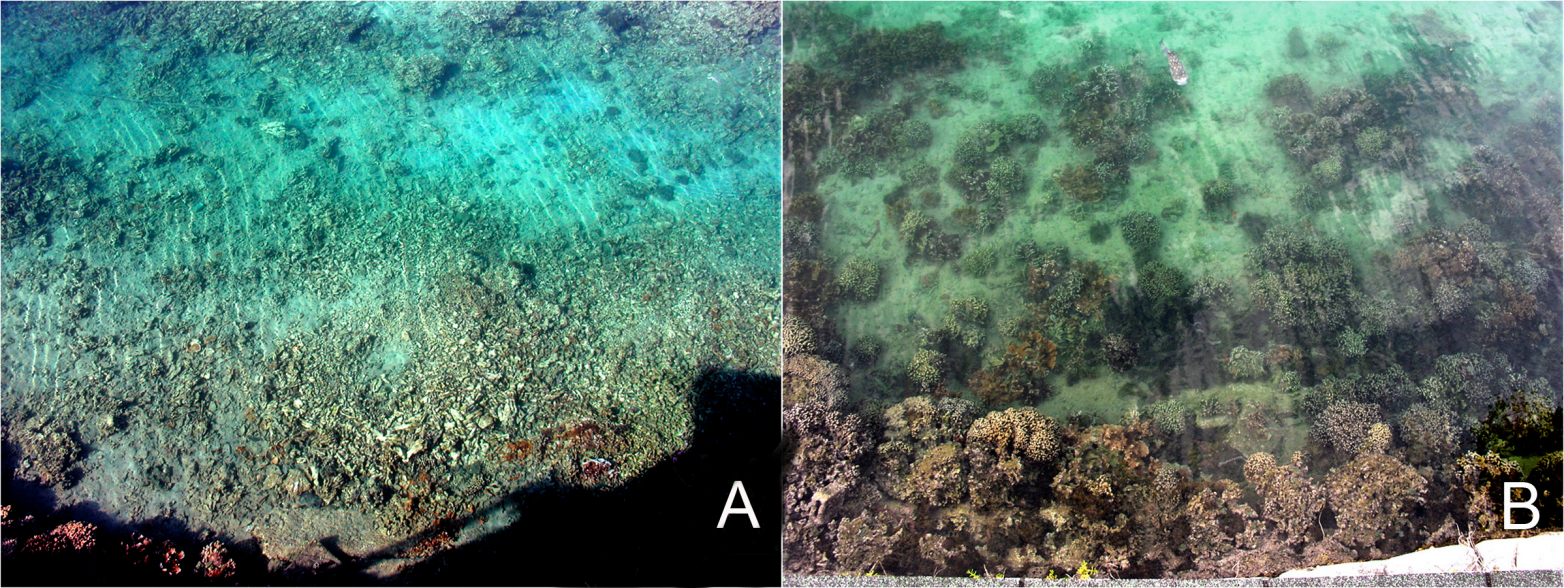
(A) Donor site after the coral was removed in 2006. (B) Coral recovery at the donor site as of 2012.

There was a significant effect of year on total coral cover in the cleared channel at the donor site (*F*_3,34_ = 9.53, *p* = 0.000, ${R}_{\mathrm{adj.}}^{2}=40.9\text{%}$) (Fig. 2). The average percent cover of all corals was the highest in 2012 (Table 1). Most of the coral increase in the channel at the donor site occurred in the first three years between 2005 and 2008 (527.5%, Table 2). Subsequently, between 2008 and 2012 there was a further increase of 57.2% in total coral cover. The differences of means between 2005 and 2008 (95% CI [0.042–0.543]) as well as 2005 and 2012 (95% CI [0.218–0.707]) were statistically significant. Between 2012 and 2016 however, total coral cover decreased by 62.7% (Table 2) and there was a significant difference of mean between these years (95% CI [−0.545–−0.070]). When differences in percent cover were analyzed by species, *M. capitata* and *P. compressa*, were not statistically significant between survey years due to high variability indicated by the coefficient of variation (Table 1).

**Table 1:** Descriptive statistics of percent cover for corals by year in the cleared channel.

Year	All corals			*M. capitata*			*P. compressa*			*n*
	Mean	s.d.	CV	Mean	s.d.	CV	Mean	s.d.	CV	
2005	4.4	4.0	90.8	1.7	1.9	116.2	2.8	3.9	140.3	9
2008	27.9	24.4	87.4	19.7	25.5	129.9	8.0	9.4	118.1	9
2013	44.3	22.8	51.6	18.1	19.8	109.5	25.9	31.1	120.2	10
2016	16.5	15.4	93.2	9.3	9.5	102.6	7.2	11.8	164.2	10

**Notes.**

s.d. one standard deviation CV coefficient of variation

**Table 2:** Changes (%) in cover between years in the cleared channel.

Year	All corals	*M. capitata*	*P. compressa*
2005–2008	527.5	91.5	65.3
2008–2013	57.2	−9.0	69.1
2013–2016	−62.7	−94.1	−259.0

### Relocation site

The invasive orange key-hole sponge, *Mycale grandis* doubled in percent cover from initial placement (1.0%) to the 2012 resurvey (2.3%).

Fish abundance increased 4,700%, mean fish length increased by over 400%, and fish biomass increased over 436,000% since the original survey in 2005. Average fish length more than tripled from 2005 to 2008 and steadily increased until 2016. Number of species has also increased with the largest increase (sevenfold) observed between 2005 and 2008. The number of species has nearly doubled since 2008. While the total number of species has increased from 2008 to 2016, the percentage of endemics in 2016 (19%) decreased since 2008 (29%). No introduced species were recorded in 2016 although in 2012 they made up 9% of the total. Herbivores absent in 2005 prior to the coral relocation currently (2016) make up over 80% of total fish at the relocation site (Table 3).

**Table 3:** Fish community structure: biomass, trophic levels, and endemism at relocation site.

Survey year	Abundance (number)	Mean length (cm)	Species richness	Herbivores (% of total)	Indigenous (% of total)	Introduced (% of total)	Endemic (% of total)	Scarids (% of total)	Biomass (Kg 100 m-2)
2016	384	12.6	12	80.2	81.3	0.0	18.8	69.5	1.27
2012	146	10.2	11	45.5	63.6	9.1	27.3	73.3	1.19
2008	148	7.4	7	57.1	71.4	0	28.6	66.9	0.71
Pre-transplant 2005	8	2	1	0	100.0	0	0	0	0.004

## Discussion

### Donor site

Erftemeijer et al. (2012) reviewed 35 documented case studies of dredged and areas near dredged coral reefs from around the world. While they acknowledged that these cases represent only a fraction of coastal development projects that affect coral reefs, the evidence shows that dredging causes turbidity and sedimentation that is very likely to result in coral cover decline (Erftemeijer et al., 2012). Large areas of dredged patch reefs may not recover because sand and fine sediment accumulation blocks coral settlement (Uchino, 2004). However, these sandy reef flats are capable of supporting a functional coral reef community if seeded with large relocated corals.

Coral transplantation has limitations and can have negative effects on donor and transplanted colonies, i.e., reduced fecundity (Szmant-Froelich, 1985; Edwards & Clark, 1999; Okubo, Motokawa & Omori, 2007) and low diversity at the transplant site (Horoszowski-Fridman & Rinkevich, 2015; Okubo & Onuma, 2015). Corals transplanted into new areas can carry flora and fauna that may disrupt the functioning ecosystem and may also render corals more susceptible to disease at the new location (Casey, Connolly & Ainsworth, 2015). These downfalls can be avoided by using larger, reproductively competent fragments or colonies, a large number of species with different life history strategies (Kotb, 2016; Darling et al., 2012), by removing any attached species that are not present at the relocation site (as in the present study), and by choosing transplantation sites that have similar environmental conditions as the donor sites (Muñoz Chagín, 1997). Lowered fecundity at restoration sites is less of a concern when relocating entire colonies; however, in these cases, donor sites are not expected to recover rapidly.

The rapid increase in coral cover at the donor site in the HIMB channel, in fact, was not anticipated. Unlike the findings by Uchino (2004), removal of the large carbonate structures and subsequent conditions in the dredged channel did not prevent recovery. Instead, rapid growth of the remaining fragments occurred (Fig. 2). Richmond & Hunter (1990) determined that asexual reproduction in habitats with unconsolidated substrata is highly important and Kolinski (2004) showed that asexual reproduction through fragmentation is the primary method of propagation by the reef coral *Montipora capitata*. The concept of rapidly increasing coral cover through spreading of fragments in Kāne‘ohe Bay (Maragos, 1974) also provides explanation for the increase observed during this study. Under the conditions of high water flushing and high irradiance found in the channel the corals would be expected to increase in radius by 1 cm to 3 cm per yr. (Jokiel & Tyler, 1993). A fragment with a radius of 2 cm in 2005 (area = 7 cm^2^) growing at a rate of 1.5 cm yr^−1^ would have increased to a radius of 6.5 cm (133 cm^2^) in 2008 for a 1900% increase in area. Our results show an overall mean increase in total coral cover of >500% between 2005 and 2008.

Since 2005, this dredged channel bottom at the donor site has served as a primary collection site for corals to be used in experiments at the marine laboratory. This was considered to be an excellent way of conserving the coral resources while keeping the channel clear. However, the amount of material removed for research purposes (1–2 m^2^) has turned out to be trivial in comparison to the documented population increase and therefore did not significantly reduce further accretion of corals in the channel.

The overall decrease (63%) in coral cover between 2012 and 2016 can be attributed to widespread bleaching events that occurred in 2014 and 2015. No bleaching was noted between 2005 and 2012, however, in 2014 an average of 45% of corals bay wide were bleached but mortality was very low (<1%). A different pattern emerged in 2015; bleached corals in the south bay, where the donor and relocation sites are located, reached 61% with significantly higher mortality (33%; Bahr, Rodgers & Jokiel, 2015). The northern and central portions of Kāne‘ohe Bay experienced high recovery after bleaching in 2014 and 2015, potentially due to their stronger connections with the open ocean but the south bay experiences much higher water residence times (1–2 months) than the northern (∼1 day) and central zones (∼6 days; Lowe et al., 2009). Inadequate nutrient exchange and transport of waste during these events likely exacerbated the bleaching situation for corals in the south bay where the donor site is located.

### Relocation site

**Figure 3:**
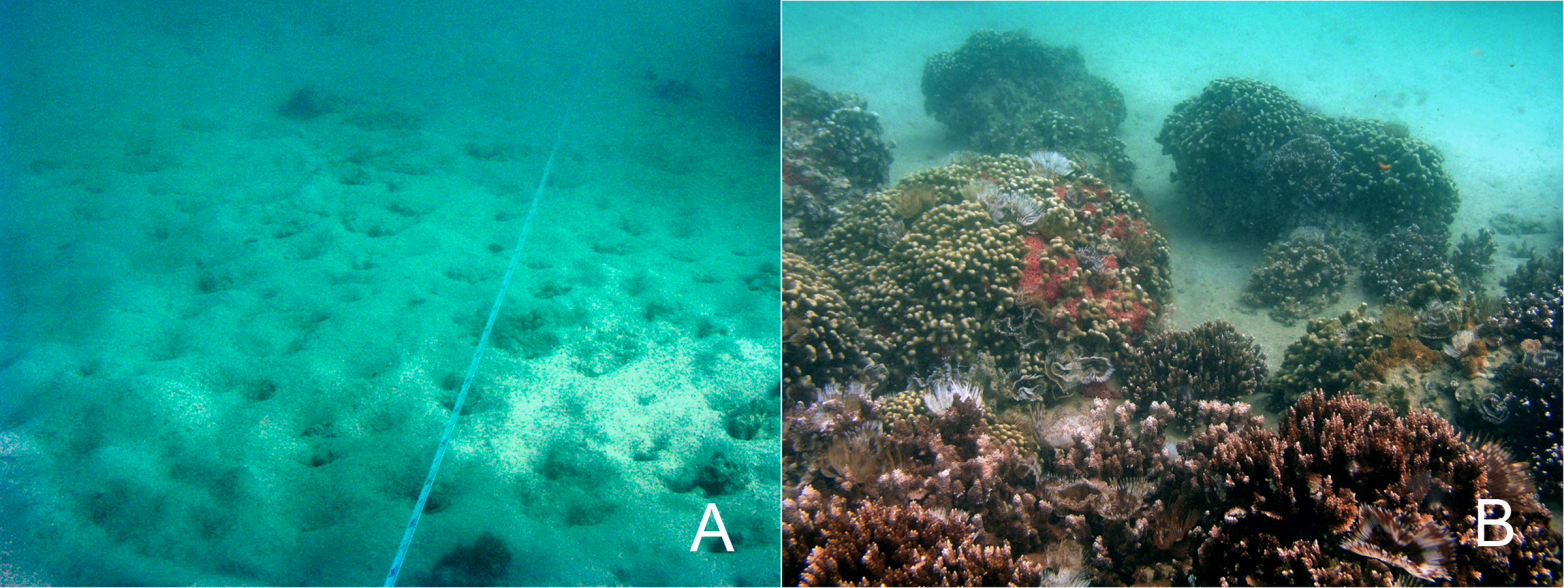
(A) Benthic habitat prior to the coral relocation in 2006. (B) Relocation site in 2012.

Ecological restoration aims to return damaged ecosystems to a healthy status. A healthy ecosystem is generally defined as one that is able to maintain structure and function while remaining resilient to stressful conditions that may occur over time (Costanza & Mageau, 1999). Healthy, resilient coral reefs are defined by a number of important ecological factors that vary greatly by location but are generally defined as having high coral diversity, habitat complexity, herbivore biomass, recruitment, tidal mixing and low nutrients and sedimentation (McClanahan et al., 2012). After the dredging of the relocation site in the late 1930s, habitat complexity was greatly diminished and the reef remained depauperate in benthic and fish species for 60 years. Following the relocation of the large coral heads, spatial complexity was greatly enhanced (Fig. 3) and the increased rugosity immediately led to an increase in numbers of fish and fish species. Fish number, biomass, and diversity are highly associated with spatial relief (Friedlander & Parrish, 1998). Large areas of barren carbonate rock on the sides of the transplanted coral colonies have been overgrown by coral tissue, further increasing bottom complexity. Increased substrate provides habitat for benthic invertebrates, which serve as the main diet of many species of fishes, which in turn are utilized at other trophic levels to further increase diversity. Habitat heterogeneity provides refuge for fishes from predation and competition and expands the availability of resources and their rate of production. Coral cover is also associated with obligate corallivores, so higher coral coverage increases corallivore populations.

The effects of successful coral transplantation have also been shown to have a positive impact on fish populations in Sulawesi, Indonesia (Newman & Chuan, 1994) and the Philippines (Shaish et al., 2010; Gomez et al., 2014) and in Tanzania (Mbije, Spanier & Rinkevich, 2013) where coral-associated invertebrates have also increased. However, fish populations are highly variable due to mobility, cryptic ability of some fishes, and diurnal shifts. A large number of transects would have to be conducted to quantify actual populations. Conversely, changes in relative populations between years can be highly evident with few transects when there are massive differences in number and biomass as in this study.

**Figure 4:**
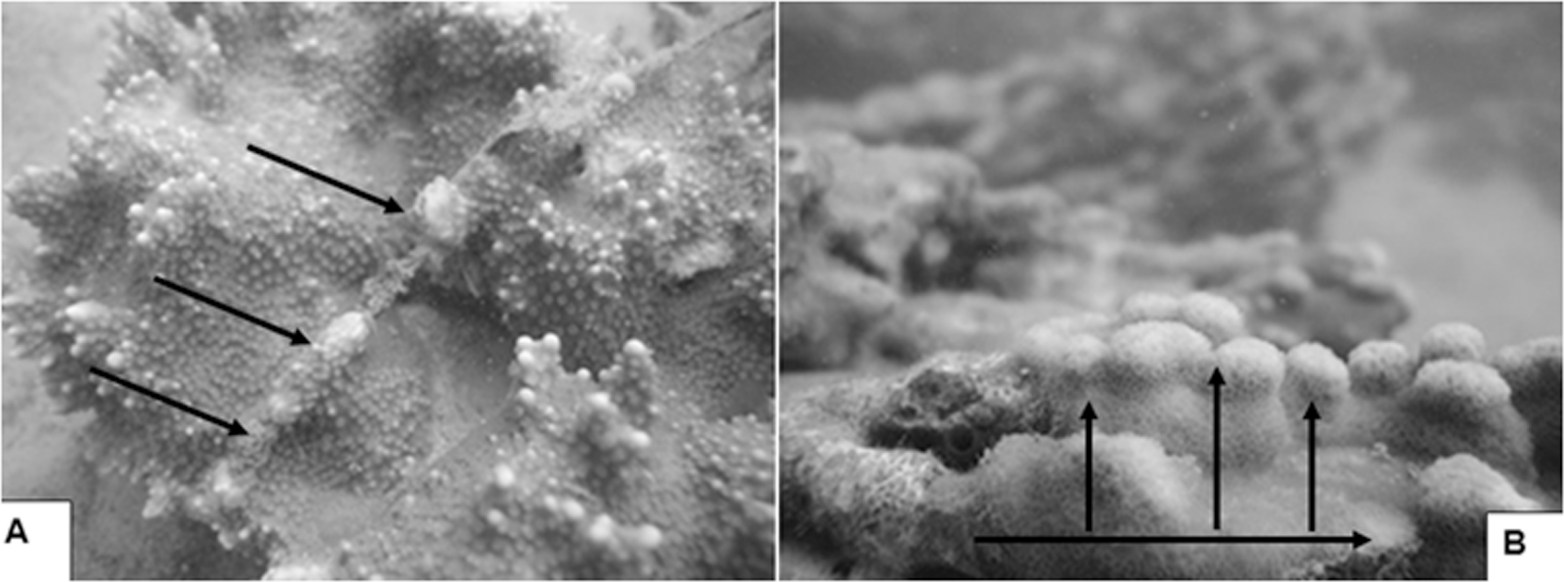
Transplanted *Montipora capitata* (A) overgrowing wire used to secure the tag. Transplanted *Porites compressa* (B) branch fragment lying on its side in sandy bottom. The horizontal arrow shows the direction of growth of the original colony prior to transplantation. The branch is sprouting many smaller vertical branches (vertical arrows) that will gradually extend to form a larger colony (April 21, 2005).

Observations of relocated corals show fragments and branches that fell on hard substratum attached to the bottom and larger colonies placed on sand were able to avoid smothering by sediment or abrasion by sand scour. Rapid coral growth is evident by overgrowth of wires used to secure tags to the corals (Fig. 4A) and large branched corals placed on their sides that changed direction of growth and formed new vertical branches (Fig. 4B). These observations are consistent with the results from other transplantation projects in Kāne‘ohe Bay (Maragos, 1974; Kolinski & Jokiel, 1996; Jokiel & Brown, 1998; Jokiel et al., 1999; Jokiel & Naughton, 2001), Mexico (Tortolero-Langarica, Cupul-Magaña & Rodríguez-Troncoso, 2014), and the Red Sea (Kotb, 2016) demonstrating transplanted corals can grow and spread across the substratum into large thickets over a period of only a few years.

Rapid reef recovery has been well documented throughout the Hawaiian Islands: in Kāne‘ohe Bay in response to sewage outfall removal (Banner, 1974; Evans, Maragos & Holthus, 1986), at Kaho‘olawe as a result of ungulate removal and soil stabilization (Jokiel, Cox & Crosby, 1993), on the island of Kaua‘i in response to high wave energy from Hurricane Iwa and Iniki (Jokiel, 2008), and on the Hamakua coast of the island of Hawai‘i that received insult from the sugar industry’s discharge of bagasse and sediment (Grigg, 1985). However, this successful relocation project is an exception when compared to most historical accounts of coral relocation in the main Hawaiian Islands and the larger Pacific region (Jokiel et al., 2006). Corals relocated nearby into favorable wave-protected areas with similar depth and environmental factors experience low mortality. Marginal habitats, however, that cannot support high coral cover will eventually suffer high initial mortality or slow decline due to wave damage, eutrophication, sedimentation or predation (Naughton & Jokiel, 2001; Jokiel, 2008). Crown of Thorns sea star predation severely reduced relocated coral colonies in Piti Bay, Guam (Kenchington & Kelleher, 1992). Stochastic wave events can abrade, displace, bury, or break corals as demonstrated at Kawaihae Harbor, Hawai‘i (Cox & Jokiel, 1995) and in Mexico (Tortolero-Langarica, Cupul-Magaña & Rodríguez-Troncoso, 2014). In other areas eutrophication can increase algal competition (Sparks, Stone & White, 2010; Banner, 1974) and sedimentation can prevent settlement, smother colonies, and block light (Jokiel et al., 2008; Erftemeijer et al., 2012). In these cases, the emphasis should be on reducing or eliminating the impact to allow natural recovery, but the major threat to relocated corals as well as to established coral reefs is from global impacts, mainly ocean temperature increase (Bahr, Rodgers & Jokiel, 2015; Ainsworth et al., 2016).

While scientists worldwide are attempting to restore currently degraded reefs a new global climate change reality is starting to become the norm. Seawater temperatures are increasing, ENSO events are having devastating effects, and degraded coral reefs are increasing in spatial extent. Coral restoration projects are better designed and implemented today than decades ago, but they may take the focus off the underlying problems. We need to reduce pollution, prevent erosion, and reduce carbon emissions. Restoration efforts on reefs vulnerable to poor land management, pollution, and/or continued severe bleaching may render restoration efforts futile. Effective translocation and management plans should include reduction or elimination of watershed stressors, establishment of marine reserves, development of integrated coastal management systems, and establishment and enforcement of regulations that protect coral reefs.

## Conclusions

This coral relocation project was highly effective in creating a thriving fish community on a barren sand/silt flat and rapid recovery of coral at the removal site. The practice of moving large coral formations from areas to be dredged or filled can be a useful management approach where conditions are suitable. The transplant mitigation strategy should be restricted to reef areas not subjected to storm surf or continuing anthropogenic impacts. The method is effective where transport distance is short and similar conditions exist at donor and receiving sites.

Implications for Practice

• Although coral recovery can be extremely rapid if conditions remain stable, stochastic events such as temperature increase can quickly reduce coral cover.
• Initial remedial coral relocation can re-establish functional coral communities.
• Transplant of mature colonies can bypass recruitment limitations and directly restore fish populations.
• Relocation of large colonies can restore resilience to otherwise marginal reefs in areas of low wave energy.
• This successful project adds to the scientific base of the emerging discipline of coral reef restoration and mitigation.
• Long-term monitoring to establish successes and failures of mitigation efforts under different conditions and in various environments are vital to improving restoration strategies.

##  Supplemental Information

10.7717/peerj.3346/supp-1 Data S1 Coral data

10.7717/peerj.3346/supp-2 Data S2 Fish data

## Additional Information and Declarations

### Field Study Permissions

The following information was supplied relating to field study approvals (i.e., approving body and any reference numbers): This research was conducted under the Hawai‘i Department of Land and Natural Resources-Division of Aquatic Resources Special Activity Permit No. 2005-25.

### Data Availability

The following information was supplied regarding data availability: The raw data has been supplied as a Supplementary File.
